# Characterization of maternal plasma biomarkers associated with delivery of small and large for gestational age infants in the MIREC study cohort

**DOI:** 10.1371/journal.pone.0204863

**Published:** 2018-11-01

**Authors:** Premkumari Kumarathasan, Gabriela Williams, Agnieszka Bielecki, Erica Blais, Denise G. Hemmings, Graeme Smith, Peter von Dadelszen, Mandy Fisher, Tye E. Arbuckle, William D. Fraser, Renaud Vincent

**Affiliations:** 1 Environmental Health Science and Research Bureau, Healthy Environments and Consumer Safety Branch, Health Canada, Ottawa, Ontario, Canada; 2 Interdisciplinary School of Health Sciences, Faculty of Health Science, University of Ottawa, Ottawa, Ontario, Canada; 3 Obstetrics and Gynecology, University of Alberta, Edmonton, Alberta, Canada; 4 Obstetrics and Gynecology, Queen’s University, Kingston, Ontario, Canada; 5 Obstetrics and Gynecology, King’s College London, London, United Kingdom; 6 Centre Hospitalier Universitaire de Sherbrooke, Sherbrooke, Québec, Canada; 7 Department of Biochemistry, Microbiology and Immunology, Faculty of Medicine, University of Ottawa, Ottawa, Ontario, Canada; Univesity of Iowa, UNITED STATES

## Abstract

**Objective:**

Neonatal morbidity and mortality can be influenced by maternal health status. Information on maternal and fetal biomarkers of adverse health outcomes is limited. This work aims at identifying maternal biomarkers associated with low and high birth weight for gestational age groups.

**Design and settings:**

Population-based prospective cohort study of the potential adverse health effects of exposure to environmental contaminants on pregnancy and infant health.

**Methods:**

Third trimester maternal plasma samples (n = 1588) from a pregnancy cohort (Maternal-Infant Research on Environmental Chemicals Study, MIREC) were analyzed for changes in a target spectrum of biomarkers of vascular health (e.g., matrix metalloproteinases MMPs, vascular endothelial cell growth factor VEGF), inflammation (e.g. cellular adhesion molecules CAMs, cytokines, chemokines) by affinity-based multiplex protein array analyses. Multivariate logistic regression analyses were done to examine associations between target plasma biomarkers, maternal-infant characteristics, and birth weight outcomes assessed as small for gestational age (SGA) ≤10^th^ percentile and large for gestational age (LGA) ≥90^th^ percentile groups.

**Results and outcomes:**

Our results revealed that maternal plasma biomarkers monocyte chemoattractant protein-1 MCP-1 (p<0.05, +ve) and VEGF (p<0.05, -ve) along with parity = 1 (p<0.01, -ve) and gestational hypertension (p<0.05, +ve) were associated with SGA births. Meanwhile, LGA was associated with maternal plasma VEGF (p<0.05, +ve) and MMP-9 (p<0.05, -ve) and gestational hypertension (p<0.01, +ve), pre-pregnancy body mass index (p<0.01, +ve), parity (p<0.05, +ve) and education (p<0.05, -ve).

**Conclusions:**

Third trimester maternal plasma biomarkers in combination with maternal health and socioeconomic characteristics can be useful in predicting SGA and LGA outcomes. Maternal vascular health and inflammatory status may contribute to both SGA and LGA births through distinct molecular mechanisms.

## Introduction

There is growing impetus to understand maternal and in-utero environmental changes that can influence both maternal and infant health [1, 2, 3]. Maternal exposures to environmental pollutants, nutritional status and life style changes including stress, are recognized as contributing factors to adverse pregnancy outcomes [4, 5, 6, 7, 8, 9, 10]. Adverse maternal and fetal outcomes encompass preeclampsia, gestational diabetes, premature rupture of membranes (PROM), preterm birth (PTB), intrauterine growth restriction (IUGR) and small and large for gestational age [11, 12, 13, 14]. Globally, adverse birth outcomes rank among the top 10 causes for disability-adjusted life year [15, 16]. There are reports on the burden of congenital affliction attributable to adverse environmental factors in Canada as well [17].

Small for gestational age (SGA) is typically considered as less than the 10^th^ sex-specific birth weight percentile for gestational age, and is commonly used as a surrogate for fetal growth restriction. SGA infants are reported to show early signs of metabolic disturbance, abnormal distribution of body fat and thus may be at risk for early onset of diabetes and heart disease [2, 3]. Also, SGA infants are more prone to have increased risk of developing neurodevelopmental disorders, including attention deficit disorder, impulsivity and autism-related disorders [18].

Large for gestational age (LGA), typically greater than the 90^th^ sex-specific birth weight percentile for gestational age is also reported to be associated with long-term health consequence [19]. For instance, macrosomia (birth weight defined as >4000 g, irrespective of gestational age) is known to be associated with several perinatal and maternal complications [20]. Large for gestational age infants are also known to develop obesity, diabetes, early-onset of cardiovascular diseases, and in addition are reported to be associated with increased future risk of cancers [21, 22].

Studies on biomarkers of maternal biological and physiological changes are evolving. Maternal biomarkers can be explored with the aim of identifying modifications in the in-utero environment that lead to various adverse birth outcomes, namely IUGR, SGA or LGA. For instance, elevated circulating levels of the vasoconstrictor peptide endothelin-1 and high blood pressure (BP) in pregnancy are related to IUGR and low birth weight [23]. Similarly, maternal hypertension is implicated in increased risk of adverse cardiovascular health in the offspring, later in childhood [24]. A recent meta-analysis suggested the need for incorporation of biophysical and maternal clinical characteristics along with biochemical markers when testing for associations with birth outcomes in order to meet the requirements of a clinically useful predictive test [25].

The goal of this work was to compare maternal plasma biomarker profiles, physiology and socio-economic characteristics of SGA and LGA live births with those that had birth weights appropriate for gestational age (AGA). For this purpose, we analysed the third trimester plasma of mothers from the Maternal-Infant Research on Environmental Chemicals (MIREC) cohort study, for a spectrum of target molecular markers representing different mechanistic pathways.

## Materials and methods

### Materials

Dulbecco’s phosphate-buffered saline (PBS, calcium and magnesium free), ethylenediaminetetra acetic acid (EDTA), diethylenetriaminepentaacetic acid (DETPA) and phenylmethylsulfonyl fluoride (PMSF) were purchased from Sigma (St. Louis, MO, USA). Butylated hydroxytoluene (BHT) was from United States Biochemical Corporation (Cleveland, OH, USA). Deionzed water (DI water) was obtained from a super-Q plus high purity water system (Millipore, Bedford, MA, USA). Antiprotease (Halt protease inhibitor) cocktail was obtained from ThermoFisher (Ottawa, ON, Canada). Multiplex kits were purchased from either Millipore (Billerica, MA, USA) or BioRad (Mississauga, ON, Canada).

### Maternal data and biospecimen collection

Third trimester (32–34 wks) maternal blood plasma samples were obtained from the MIREC Study cohort described by Arbuckle et al. (2013) [26]. In brief, between 2008 and 2011, 1983 women were recruited during their first trimester of pregnancy from ten cities in six provinces across Canada and followed each trimester until delivery. Information on the pregnancy, infant birth weight and gestational age of singleton live births were extracted from the medical charts. Questionnaires administered by trained staff included socio-demographic data, medical history and life style.

### Ethics

The research protocol, questionnaires, consent forms and recruitment posters and pamphlets were reviewed and approved by human studies research ethics committees, including the Research Ethics Board at Health Canada and the ethics committee at the coordinating center at CHU Sainte-Justine in Montreal, as well as ten other academic and hospital ethics committees across Canada. The ten other research ethics boards were: University of British Colombia—Children’s and Women’s Health Centre of BC Research Ethics Board; University of Alberta Health Research Ethics Board; University of Manitoba, Bannatyne Campus Research Ethics Board; Toronto, Mount Sinai Hospital Research Ethics Board; Hamilton Health Science/McMaster Research Ethics Board; Sudbury General Hospital, Health Sciences North Research Ethics Board; Kingston General Hospital, Queen’s Universtiy Research Ethics Board; The Ottawa Hospital Research Ethics Board; Montreal, Jewish General Hospital Research Ethics Office; and Halifax, IWK Health Centre Research Ethics Board. The Research Ethics Board (REB) approval date was August, 22, 2006. The REB reference number was 2006–0027. All participants signed informed consent forms.

### Plasma sample preparation

Plasma samples (n = 1588) were treated with DETPA, BHT and antiprotease cocktail, vortexed, and were frozen for storage until analyses of target plasma biomarkers of interest following previously reported procedures [27, 28].

### Affinity-based multiplex protein array analyses

Maternal plasma samples were analyzed for target biomarkers, namely cytokines [interleukins (ILs), tumor necrosis factor-alpha (TNF-α), interferon gamma (IFN-γ), granulocyte-macrophage colony-stimulating factor (GMCSF)], chemokines [monocyte chemoattractant protein-1(MCP-1), macrophage inflammatory protein-1 beta (MIP-1β)], vascular endothelial growth factor (VEGF-A), acute phase proteins C-reactive protein (CRP), soluble intracellular adhesion molecule (ICAM-1), soluble vascular cell adhesion molecule (VCAM-1) and matrix metalloproteinases (MMPs) by affinity-based multiplex protein array assays using Bio-Plex Pro Human panels (Bio-Rad, Canada) and Milliplex Map kits (Millipore, Canada). Briefly, plasma samples were incubated with capture antibody-coated magnetic beads, then washed and reacted with biotinylated-detection antibodies followed by incubation with streptavidin-phycoerythrin. The bead complex was washed and re-suspended in sheath fluid (Bio-Rad, Canada) and analysed using a Bioplex 100 instrument with Bioplex Manager 6.0 software (Bio-Rad, Canada).

### Statistical analyses

Statistical analyses were restricted to pregnant women from the MIREC study who had singleton live births (with birth weight recorded) and provided a blood sample during their third trimester of pregnancy (n = 1,578). Our analysis focused on a spectrum of nineteen maternal third trimester plasma target biomarkers implicated in oxidative stress, inflammation and vascular function, and their relationship with small and large for gestational age (SGA/LGA) outcomes. Newborns were defined as small for gestational age (SGA), large for gestational age (LGA) or appropriate for gestational age (AGA) based on the birth weight percentile for gestational age reference values reported on the Canadian population study by Kramer et al. (2001) [29], taking the sex of the infant into consideration. Gestational age was determined based on the women’s recall of the date of her last menstrual period, except if it differed from the date given by an early ultrasound by more than a week, in which case the date estimated by the ultrasound was taken.

Biomarker levels were compared across recruitment sites to ensure that no bias by recruitment site existed. Given the large percentage (28%) of missing values for gestational diabetes, this variable was not included in the analyses. Any plasma marker results below the limits of detection (LOD) were substituted with LOD/2. Maternal characteristics were chosen based on their known relationships with SGA and LGA, and their availability from the MIREC datasets. These included education (high school diploma or less, college/trades diploma, completed university degree), ethnicity (white, otherwise), marital status (married/common law/partner, otherwise), and smoking status (never/previous smoker, stopped during pregnancy/current smoker). Gestational hypertension and preeclampsia (GH) were based on the Society of Obstetricians and Gynaecologists of Canada (SOGC) guidelines, as follows: an elevated average systolic and/or diastolic blood pressure at >20 weeks gestation; a diagnosis of GH; taking antihypertensive therapy after >20 weeks gestation; or an elevated systolic and/or diastolic blood pressure after admission for delivery on two separate measurements taken 4 hours apart (women were excluded from this category if their high blood pressure was only during labour and they had no diagnosis of GH, were not taking hypertensive medication and had no evidence of proteinuria). The category of household income was based on the income threshold (ca. $80000/year, otherwise) for the Canadian two-parent family [30]. Caffeine (cups per day) and alcohol (drinks per day) consumption were categorized according to the questionnaire measures: caffeine from coffee and tea; one drink of alcohol is equivalent to 8 oz of beer, 4 oz of wine, and 1 oz of spirits.

Initially, descriptive statistics and plots were done on maternal plasma biomarkers to illustrate the distribution of data (not shown). For the analysis of associations with birth outcomes, biomarker data were log-transformed due to their positively skewed distributions. Normality tests were satisfactory following these transformations. Subsequently, univariate analyses using simple logistic regression and contingency tables were done to examine the distribution of continuous variables with respect to the different birth weight for gestational age groups (Table 1). The relationship between categorical variables and SGA or LGA birth outcome was examined by bivariate analyses (Pearson’s chi-square at the 5% significance level), using unadjusted odds ratios as an approximation of risk ratios (Table 2). Low incidence impeded the inclusion of covariates such as second-hand smoke and individual chronic conditions (e.g., pre-pregnancy hypertension, asthma).

**Table 1:** Descriptive statistical analysis results for maternal variables (continuous data) in relation to SGA, AGA and LGA births.

	LOD (mg/ml)	n	<LOD (%)	range	μ	SD (μ)	SGA ^1^ (10^th^ percentile)				AGA ^1^ (10^th^—90^th^ percentile)				LGA ^1^ (90^th^ percentile)			
							range	μ	SD (μ)	sig	range	μ	SD (μ)	sig	range	μ	SD (μ)	sig
**BIOMARKERS**																		
CRP(pg/ml)	0	1,578	0	[108,1602600]	17,290.61	435.24	[562,184192]	18,952.52	1,965.28		[358,1602600]	32519.03	67536.66		[108,655732]	17,803.25	1,246.48	
ICAM	2.4	1,581	0	[1266,577276]	143,922.31	3,619.61	[32665,318178]	154,382.97	16,095.54		[1266,577276]	160024.53	68221.69		[32203,400681]	147,690.91	10,340.43	
VCAM	0.6	1,586	0	[42262,711948]	250,701.53	6,295.14	[102587,494366]	265,886.79	27,571.18		[42262,711948]	263453.96	90698.57		[81795,594883]	262,796.97	18,399.47	*
VEGF	0.2	1,587	2	[0.02,47]	2.33	0.06	[0.09,23]	1.73	0.18	*	[0.02,29]	3.62	3.61		[0.08,47]	2.96	0.21	**
MMP_1	3	1,588	0	[44,16436]	706.41	17.73	[131,4158]	775.15	80.38		[66,12215]	930.77	911.75		[44,16436]	704.05	49.29	
MMP_2	200	1,564	0	[6097,6803100]	67,034.52	1,695.04	[30024,5952200]	73,818.70	7,654.64		[6097,6803100]	96958.73	384520.12		[8531,204490]	61,818.85	4,360.37	*
MMP_7	97	1,564	0	[142,64732]	5,772.02	145.95	[7212,20705]	5,776.24	598.97		[212,64732]	7105.73	4712.85		[142,39660]	5,471.55	385.93	
MMP_9	2	1,588	0	[4376,2060700]	28,548.06	716.39	[5989,2060700]	30,782.97	3,192.04		[4376,1532600]	51030.87	120718.99		[6132,436164]	26,351.49	1,844.97	
MMP_10	5	1,557	0	[21,3281]	261.69	6.63	[64,891]	277	28.88		[21,3281]	300.15	199.14		[82,1039]	243.71	17.19	*
GMCSF	0.15	1,583	3	[0.01,219]	1.21	0.03	[0.18,24]	1.34	0.14		[0.01,218]	2.56	7.79		[0.01,718]	1.07	0.08	
MIP_1B	2.4	1,587	0	[8,469]	57.84	1.45	[26,144]	61.25	6.35		[8,469]	61.77	27.08		[25,285]	59.45	4.16	
IFNG	0.18	1,583	1	[0.05,204]	4.16	0.1	[0.2,35]	5.03	0.52		[0.05,204]	8.53	16.74		[0.11,109]	3.9	0.27	
MCP_1	1.1	1,588	0	[6,230]	38.06	0.96	[13,230]	42.41	4.4	*	[7,213]	42.63	22.16		[6,133]	36.98	2.6	
TNFA	0.07	1,574	0	[0.2,133]	4.36	0.11	[1,11]	4.34	0.45		[0.2,133]	5	4.51		[1.14,18]	4.4	0.31	
IL_2	0.26	1,586	14	[0.01,216]	1.1	0.03	[0.04,216]	1.14	0.12		[0.01,216]	3.35	10.5		[0.01,95]	1.02	0.07	
IL_6	0.2	1,583	1	[0.07,185]	1.73	0.04	[0.19,9]	1.73	0.18		[0.07,185]	2.94	7.51		[0.22,57]	1.87	0.13	
IL_8	0.05	1,583	0	[0.18,40]	2.06	0.05	[0.47,7]	2.06	0.21		[0.2,40]	2.57	2.53		[0.25,15]	2.04	0.14	
IL_10	0.48	1,587	<1	[0.10,721]	21.03	0.53	[4,331]	21.36	2.21		[0.1,656]	31.06	49.18		[0.84,721]	21.99	1.54	
IL_12	0.34	1,583	7	[0.01,461]	2.29	0.06	[0.02,229]	2.67	0.28		[0.01,461]	7.72	26.21		[0.02,225]	2.14	0.15	
**CHARACTERISTICS AND DELIVERY**				**range**	**average**	**SD**	**range**	**average**	**SD**		**range**	**average**	**SD**		**range**	**average**	**SD**	
Pre-pregnancy BMI (kg/m^2^)				[15.6,59]	24.86	5.5	[18,39]	24.08	4.73		[15.6,55.3]	24.69	5.43		[18,59]	26.34	6.04	*
Maternal age (calendar years)				[19,49]	33.18	5.01	[19,44]	33	5.26		[20,48]	33.17	4.96		[19,49]	33.29	5.25	
Caffeine consumption (cups^2^/day)				[0,10]	0.72	0.87	[0,3.43]	0.72	0.77		[0,8]	0.71	0.85		[0,10]	0.72	1.02	
Alcohol (drinks ^3^/day)				[0,0.5]	0.01	0.04	[0,0.17]	0.01	0.03		[0,0.50]	0.01	0.04		[0,0.36]	0.01	0.03	
Gestational age (completed weeks)				[32,42]	39.45	1.42	[34,42]	39.59	1.47		[32,42]	39.44	1.41		[33,42]	39.42	1.42	

Notes: LOD = limit of detection, measured in pg/ml, except for CRP (ng/ml); μ = geometric mean; SD (μ) = standard deviation of the mean;

* p < 0.05;

** p < 0.01.

^1^ Small for Gestational Age (SGA) and Large for Gestational Age (LGA) are in completed weeks. Reference category for each is Average for Gestational Age (AGA): 10th<AGA<90th percentile.

^2^ Caffeine consumption from coffee and other caffeinated drinks.

^3^ Alcoholic drinks measured as: beer = 8 oz, wine = 4 oz, and spirits = 1 oz.

**Table 2:** Results of statistical analyses for categorical maternal and infant variables.

	*Sample*		*Small for Gestational Age (SGA)*							Appropriate for Gestational Age (AGA)						Large for Gestational Age (LGA)						
	*N = 1*,*578*		*N*_*S*_ = *93*							*N*_*A*_ = *1*,*281*						*N*_*L*_ = *204*						
	*(n)* ^1^	*(%)* ^1^	*(n*_*S*_*)* ^1^	n_S_/(n-n_L_)(%) ^1^	OR	95% CI	p-value	sig		(n_A_) ^1^	n_A_/n(%) ^1^	OR	95% CI	p-value	sig	(n_L_) ^1^	n_L_/(n-n_S_)(%) ^1^	OR	95% CI	p-value	sig	
**BABY**																						
**Gender**																						
Boys (R)	*828*	*52*	*50*	7	1					682	82	1				96	12	1				
Girls	*749*	*47*	*43*	7	1	[0.64–1.49]	0.8			598	80	0.85	[0.66–1.09]	0.2		108	15	1.3	[0.95–1.72]	0.1		
**SOCIO-DEMOGRAPHIC CHARACTERISTICS**																						
**Maternal education**																						
High school diploma or less(R)	214	14	60	30	1					138	64	1				16	10	1				†
College/trades diploma	369	23	18	6	0.1	[0.08–0.25]	<0.001	***		299	81	2.35	[1.60–3.44]	<0.001	***	52	15	1.5	[0.83–2.70]	0.18		
University degree	993	63	15	2	0	[0.02–0.07]	<0.001	***	†	843	85	3.1	[2.23–4.31]	<0.001	***	135	14	1.4	[0.80–2.37]	0.25		
**Ethnicity**																						
White (R)	1,293	82	73	7	1					1,050	81	1				170	14	1				
Non-white	285	18	20	8	1.3	[0.75–2.09]	0.4		†	231	81	0.99	[0.71–1.37]	0.95		34	13	0.9	[0.61–1.35]	0.64		
**Marital status**																						
Married/common law (R)	1,505	95	85	7	1					1,222	81	1				198	14	1				
Otherwise	73	5	8	12	2	[0.91–4.20]	0.08		†	59	81	0.98	[0.54–1.78]	0.94		6	9	0.6	[0.27–1.47]	0.28		†
**Household income**																						
< $80,000 (R)	592	38	49	9	1					472	80	1	[0.91–1.55]	0.19		71	13	1				
$80,000 +	917	58	37	5	0.5	[0.30–0.73]	<0.001	***		756	82	1.19	[0.46–1.52]	0.57		124	14	1.1	[0.80–1.49]	0.59		
**FAMILY AND HEALTH HISTORY**																						
**Parity** ^2^ **[0 to 7]**																						
0 (R)	699	44	60	10	1					560	80	1				79	12	1				
1	639	40	21	4	0.4	[0.23–0.63]	<0.001	***	†	520	81	1.08	[0.82–1.42]	0.56		98	16	1.3	[0.97–1.84]	0.08		
2+	240	15	12	6	0.6	[0.30–1.06]	0.07		†	201	84	1.18	[0.80–1.74]	0.22		27	12	0.7	[0.45–1.13]	0.84		†
**Reported diabetes**																						
No (R)	1,560	99	-	-						1,270	81	1				198	13	1				
Yes	18	1	-	-						11	61	0.36	[0.14–0.94]	0.03	*	6	35	34	[1.30–9.43]	0.01	**	†
**Reported chronic conditions other than hypetension/diabetes**																						
No (R)	1,230	78	65	6	1					1,009	82	1				156	13	1				
Yes	358	23	28	9	1.5	[0.97–2.44]	0.06		†	282	79	0.81	[0.60–1.08]	0.16		48	15	1.1	[0.78–1.56]	0.72		
**Prenatal multivitamin preparation**																						
No (R)	197	12	8	5	1					168	85	1				21	11	1				
Yes	1,381	88	85	7	1.6	[0.76–3.36]	0.21			1,113	81	0.72	[0.47–1.09]	0.12		183	14	1.3	[0.82–2.13]	0.31		
**Folic acid supplements**																						
No (R)	1,070	68	62	7	1					869	81	1				139	14	1				
Yes	495	31	31	7	1.1	[0.69–1.69]	0.73			402	81	1	[0.76–1.31]	1		62	13	1	[0.70–1.32]	0.8		
**GESTATIONAL-RELATED HEALTH**																						
**Gestational hypertension**																						
No (R)	1,430	91	76	6	1					1,181	83	1				173	13	1				
Yes	148	9	17	15	2.6	[1.50,4.64]	<0.001	***		100	68	0.44	[0.30–0.64]	<0.001	***	31	24	2.1	[1.37–3.26]	<0.001	**	

Note: (R) = reference category; OR = odds ratio; CI = confidence interval (95%);

* p < 0.05;

** p < 0.01;

*** p < 0.001;

ns = not significant

^1^ May not add to 100% due to rounding. SGA percentages do not include LGA, and vice versa (LGA percentages do not include SGA).

^2^ Parity ranges from 0 (no previous live births) to 7; and 0 to 3 for SGA and LGA. Above, parity of two or more are collapsed due to confidentiality.

^†^ Estimate’s coefficient of variation (CV) is between 16.7% and 33.3%. Estimates should be interpreted with caution.

In the second part of our analyses, to test the association between all maternal plasma biomarkers and birth weight outcomes using multivariate models, we used a 20% significance level for inclusion of the covariates, as a variable-reduction method. (Table 3) AGA was the reference group in all analyses. We modelled the probability of the infant being SGA or LGA with a forward logistic regression, using Firth’s correction. This is a penalized likelihood estimate due to low frequency of one of the outcome variables (SGA with 93 newborns). Although LGA would be considered “large enough” with 204 newborns, Firth’s estimates would be nearly the same as the unconditional estimates [31]. The latter was confirmed for our study (comparison not shown). All analyses were conducted using PROC FREQ and PROC LOGISTIC in SAS/STAT software, version 5.1 of SAS EG for Windows (SAS Institute Inc., Cary, NC) and the function LOGISTF in Rsoftware, version 3.1.1 for PC (The R Foundation for Statistical Computing).

**Table 3:** Multivariate logistic regression analysis results exhibiting the association between maternal parameters and the odds for adverse birth outcomes.

	Small for Gestational Age (SGA)^1^								Large for Gestational Age (LGA)^1^							
	unadjusted				adjusted				unadjusted				adjusted			
	β	OR	CI (95%)		β	OR	CI (95%)		β	OR	CI (95%)		β	OR	CI (95%)	
Intercept					-4.2	0	[.005, .04]	***					-1	0.4	[0.04, 3.00]	
**Biomarkers**																
VEGF	-0.2	0.8	[0.65, 0.94]	*	-0.2	0.8	[0.71, 0.92]	*	0.25	1.3	[1.10, 1.48]	**	0.25	1.3	[1.10, 1.51]	**
MCP-1	0.48	1.6	[1.04, 2.49]	*	0.5	1.7	[1.22, 2.21]	*							--	
MMP-9							--		-0.1	0.9	[0.74, 1.06]		-0.2	0.8	[0.66, 0.98]	*
**Maternal health**																
Parity (1 previous birth)					-0.7	0.5	[0.34, 0.66]	**					0.33	1.4	[1.01, 1.91]	*
Gestational hypertension					0.73	2.1	[1.39, 3.01]	*					0.67	1.9	[1.20, 3.07]	**
Pre-pregnancy BMI							--						0.04	1	[1.01, 1.07]	**
**Socio-economic characteristics**																
Education (College/trades diploma)							--						-0.4	0.7	[0.44, 0.98]	*

Note: OR = odds ratio; CI = confidence interval, α = 5%;

* p < 0.05;

** p < 0.01;

*** p < 0.001.

^1^ SGA percentages do not include LGA, and vice versa (LGA percentages do not include SGA). Wald test for SGA model = 25.78*** (4 d.f.) and for LGA model = 39.83*** (6 d.f.)

## Results

In our study, 93 (5.9%) newborns were identified as SGA and 204 (12.9%) as LGA. There were 828 boys (52.5%) and 749 girls (47.5%). The majority of mothers had completed college, trades or post-secondary education (86.4%), were white (81.9%), married or in common-law (95.4%), and their yearly household income was at least $80,000 (58.0%). There were 699 (44.3%) first-time mothers, most reported taking multi-vitamin preparations (87.5%), and 22.7% reported having chronic conditions (22.7%).

Descriptive statistics results on the distribution patterns (range, geometric or arithmetic mean, and standard deviation) for plasma biomarkers and maternal characteristics (for continuous data) are provided in Table 1. Detection levels for the maternal plasma biomarkers were high, ranging from 100% to 86%. The mean pre-pregnancy BMI for these women was below the classification for obesity (BMI ≥ 30kg/m^2^). Average maternal age at delivery was 33 years and average GA at delivery slightly under 40 weeks. Caffeine and alcohol consumption were rare events (average of 0.72 cups/day and 0.01 drinks /day, respectively).

In the univariate analysis, significant associations (p<0.05) with SGA and LGA are also included in Table 1: For instance, the association of MCP-1 with SGA was positive. VEGF was found to be negatively associated with SGA but positively associated with LGA (p<0.001). Maternal plasma VCAM-1 was positively associated with LGA, while MMP-2 and MMP-10 were negatively associated with LGA. Pre-pregnancy BMI was found to be positively associated (p<0.05) with the LGA group.

Table 2 illustrates the distribution of categorical variables, and their association with SGA and LGA. Higher maternal education, higher household income, and having one previous live birth (p<0.001) decreased the risk of SGA births. Gestational hypertension increased the risk of both SGA (p<0.001) and LGA (p<0.01). Reported diabetes also increased the risk of LGA (p<0.01) births.

The results of our multivariate analyses for the relationship between maternal parameters and SGA, LGA outcomes are provided in Table 3. Increased VEGF levels and parity = 1 decreased the odds for SGA: a unit increase in VEGF levels in the third trimester resulted in a 19% decrease in the odds of SGA; having had one previous live birth resulted in a 52% decrease in SGA births. Conversely, a unit increase in MCP-1 was related with a 65% increase in the odds of SGA, while gestational hypertension increased the odds over 2-fold (106%). Furthermore, a unit increment in VEGF increased the odds for LGA babies by 28%, while a unit increase in MMP-9 decreased the odds for LGA births by 20%. In terms of maternal health and physical characteristics, gestational hypertension, parity = 1, and an increase in pre-pregnancy BMI were associated with increased odds for LGA births by 94%, 39%, and 4%, respectively, and increased maternal education resulted in a 34% decrease in the odds of delivering an LGA infant.

## Discussion

### Main finding and interpretation

Our preliminary work conducted on maternal plasma biomarkers from small subsets of the MIREC study subjects suggested that disruption in mechanisms associated maternal vascular function or immune health status may influence infant birth weight outcomes [28, 32]. Also, studies have shown that prenatal exposure to environmental chemicals can lead to maternal biological changes and changes in birth outcomes [33, 7, 1, 34, 35]. Examination of the relationship between maternal biomarker changes and SGA or LGA births in the MIREC mother-infant cohort can be informative in terms of gaining information on predictors of these birth outcomes.

Our current findings from the MIREC cohort revealed a wide range of plasma biomarker concentrations among the MIREC mothers, during the third trimester. Alcohol consumption and smoking were minimal for the participants from this study. Small and large for gestational age cases in this mother-infant cohort were about 5.9 and 12.9%, respectively, compared to 8.3 and 10.4% in the 2010 general Canadian birth population (Perinatal Health Indicators for Canada 2013 http://www.phac-aspc.gc.ca/rhs-ssg/phi-isp-2013-eng.php).

The descriptive statistics results identified a negative association (p<0.05) between maternal third trimester plasma VEGF-A and SGA (Table 1). The vascular endothelial growth factor family consist of key molecules including VEGF-A, placental growth factor (PlGF), and the receptors VEGF receptor 1 (FLT-1) and VEGF receptor 2 (KDR) that are implicated in angiogenesis and vasculogenesis for early placental development and vascularization [36, 37, 38]. Moreover, a splice variant of FLT-1 known as soluble FLT-1 (sFLT-1) is known to play an antagonistic role compared to VEGF-A and PlGF. sFLT-1 has high binding affinity towards VEGF-A, [39] and this can result in low circulating VEGF-A levels [40]. There are reports of reduced serum VEGF-A and PlGF in pre-eclampsia [41, 42], and injection of adenovirus expressing sFLT-1 into the tail vain led to pre-eclampsia symptoms in rats [43]. PlGF has been associated with fetal growth restriction (FGR) caused by placental dysfunction, and maternal PlGF levels were shown to discriminate fetuses with placental IUGR from constitutionally small foetuses [44, 45].

Meanwhile, plasma MCP-1 was positively associated with SGA births. (Table 1) Increased plasma MCP-1 is associated with stress factors and infection during pregnancy [46]. Also, MCP-1gene expression is increased in the placenta of women infected with malaria during pregnancy that results in low birth weight infants [47]. Similarly, the matrix metalloproteinases, MMP-2 and MMP-10, as well as VCAM-1 and VEGF exhibited strong associations with the LGA outcome (Table 1). Matrix metalloproteinases play a role in implantation, and are regulated by growth factors, cytokines and reproductive hormones [48]. MMP-2 levels are increased in the circulation of women who later developed preeclampsia [49]. VCAM-1 is a cellular adhesion molecule, and is a biomarker of endothelial dysfunction [50, 51], and interestingly it was lower (p<0.05) in the LGA group, compared to the other groups (Table 1). Among the maternal parameters, pre-pregnancy BMI, education, household income, parity, reported diabetes, and gestational hypertension were associated with either SGA or LGA birth outcomes. (Tables 1&2) These results are in line with previous findings, especially the influence of maternal BMI and pregnancy-induced hypertension on birth outcomes [52, 53, 54, 55].

We employed a reductionist approach for the multivariate model analyses to identify potential maternal determinants of adverse birth outcomes with the adjustment for a rich set of covariates, while obtaining a parsimonious model (Table 3). Our findings identified maternal plasma VEGF, MCP-1, parity and gestational hypertension to be adequate to predict SGA births, based on the best fit model. In terms of third trimester maternal plasma biomarkers, higher VEGF levels were associated with a lower (p<0.05) odds ratio for SGA births, while higher plasma MCP-1 was associated with an elevated odds ratio for SGA infants (Table 3). Also, having had one previous birth (parity = 1) reduced the odds for SGA, while gestational hypertension increased the odds for this adverse birth outcome. In the mothers with SGA infants, it is plausible that decreased circulating VEGF-A levels may be due to increased sFLT-1 levels as seen with preeclampsia which may constitute a part of SGA cases in this study. This is also supported by the directionality of gestational hypertension in the model, for the SGA group (Table 3). Increased sFLT-1 levels in rats have been associated with hypertension and proteinuria characteristic of preeclampsia [43]. In addition, since VEGF-A is known to promote vasodilation by mediating endothelial NO synthesis [38], low VEGF-A levels may adversely affect vasodilation mechanisms and perhaps lead to reduced utero-placental perfusion and SGA outcome. Moreover, increased third trimester maternal MCP-1 may be associated with increased neutrophil activation and perhaps enhanced proinflammatory pathways as with the conditions during spontaneous term and preterm labour [56]. The activation of proinflammatory pathways can also be conceived by the assessment of results in Table 1 which shows that mean VCAM-1 (acute phase protein) levels are relatively higher in mothers with SGA infants, compared to the other groups. Increased circulating VCAM-1 levels are observed in inflammatory conditions as well as with endothelial dysfunction [50, 51, 57]. Furthermore, an index of inflammatory condition [58] expressed as a ratio of the sum of pro-inflammatory biomarkers to IL-10 an anti-inflammatory biomarker ((MIP-1β_+_ IFN-γ_+_ TNF-α_+_ MCP-1_+_IL-2_+_ IL-6_+_ IL-8)/(IL-10), using the mean values from Table 1) revealed that this index was larger for the SGA group (5.5) compared to the AGA group (4.1), supporting the notion of a proinflammatory status in the mothers of the SGA infants. These findings are in line with previous reports [28, 32, 56]. Interestingly, administration of the anti-inflammatory cytokine IL-10 in animal models has been shown to restore birth weight and prevent preterm birth [46].

Our results also showed that the LGA births were associated with increased third trimester maternal plasma VEGF, decreased MMP-9 levels, positively associated with gestational hypertension, pre-pregnancy BMI, and parity (one previous birth) and negatively associated with maternal education. In contrast to the SGA outcome, the association of VEGF with the LGA outcome is positive, suggesting perhaps increased endothelial NO production thus favouring vasodilation pathways, and thus potentially enhanced utero-placental perfusion [59]. The negative association of LGA birth outcome with third trimester plasma MMP-9 levels suggests that in these mothers, mechanistic changes underlying the preparation for parturition in this trimester is less activated compared to the AGA group. High maternal plasma MMP-9 levels are associated with spontaneous preterm birth [60]. In our previous work, we observed reduced infant birth weights with increased circulating maternal MMP-9 levels [32]. Tightly controlled proinflammatory responses are essential in the uterus for the retention of the fetus [46]. However, cervical dilation for parturition requires heightened proinflammatory responses. Moreover, the positive association between LGA outcome and pre-pregnancy BMI as well as gestational hypertension in this study is consistent with previous findings [52]. Although the LGA mothers appeared to be associated (p<0.05) with increased gestational hypertension, BMI and reported diabetes (Tables 1–3), circulating VEGF levels were high, more likely supporting angiogenesis and NO-mediated vascular effects. In addition, the inflammatory index analysis showed that the LGA group is associated with higher inflammatory index (5.0) compared to the AGA group, suggesting a proinflammatory status in LGA mothers as found in the SGA mothers. Furthermore, maternal education was negatively associated with the LGA outcome in this cohort, but not with the SGA births.

Based on our findings, it appears that different inflammatory and vascular pathways may be operative in maternal/fetal programming for SGA/LGA birth outcomes [32]. Since we see a positive association between gestational hypertension and both SGA and LGA outcomes, it is possible that the underlying mechanistic features of these birth outcomes may be similar to that found in preeclampsia. Decreased utero-placental perfusion and reduced birth weight is implicated in the etiology of preeclampsia, nevertheless, preeclampsia can also be associated with high birth weight for gestational age [61, 62, 63, 64, 65]. Our findings are consistent with previous reports, and imply that the heterogeneity observed in adverse birth outcomes could be due to the manifestation of distinct vascular effects and inflammatory mechanisms.

### Strengths and limitations

The advantages of this study include the prospective design, large mother-infant cohort, measurements of various mother-infant parameters obtained by trained professionals and the rich set of covariates. To our knowledge, this is the first work that has integrated the third trimester maternal plasma biomarkers in a large Canadian mother-infant cohort with maternal socioeconomic status, and maternal physiological parameters (e.g blood pressure) as an intermediate outcome to investigate the adverse pregnancy outcomes SGA and LGA. Our findings suggest VEGF-related vascular mechanisms, neutrophil recruitment-related inflammatory mechanisms and extracellular matrix remodelling pathways roles in precipitating SGA, LGA outcomes, with clear differences in mechanistic pathways for these adverse birth outcomes. However, due to consideration of parsimony in the multivariate statistical analyses used in this study, the mechanistic information is not visible. Also, the small subset of plasma biomarkers that reached significance in the best fit model for the SGA/LGA outcomes (Table 3) may act as surrogates for other mechanistically relevant plasma biomarkers, and can serve as tags to identify mechanistic details through future high content maternal plasma biomarker analyses. This work therefore warrants future high-content maternal biomarker analyses following a systems biology approach to resolve SGA/LGA phenotypes, and to understand the mode of action of maternal factors such as prenatal environmental chemical exposures.

The MIREC study population is not representative of the Canadian population at large, since the study was based on a convenience sample and participants were generally above the national average in terms of income and education [26]. Certain health conditions and lifestyle habits that were known to be associated with SGA and LGA, such as pre-pregnancy hypertension, asthma, smoking, and alcohol consumption were excluded due to low frequencies. Also, some potential confounders such as paternal physical characteristics were not captured in this study.

## Conclusion

Our findings suggest that perturbation in vascular function, activation of pro-inflammatory pathways and changes in extracellular matrix remodelling in mothers during the third trimester of pregnancy can be some of the determining factors of SGA or LGA births. These results also reveal that although inflammatory cascades and vascular biochemistry may contribute to adverse birth weight outcomes, pathway-specificity can be critical in defining these different phenotypes. Future focus on high-content maternal proteomic, metabolomic and epigenetic biomarkers can add value by increasing the sensitivity and specificity of such analyses to distinguish these phenotypes of adverse birth outcomes, based on comprehensive molecular mechanisms.
